# Antibiotic-Loaded Platelet-Rich Fibrin (AL-PRF) as a New Carrier for Antimicrobials: A Systematic Review of In Vitro Studies

**DOI:** 10.3390/ijms26052140

**Published:** 2025-02-27

**Authors:** Wojciech Niemczyk, Jacek Żurek, Stanisław Niemczyk, Małgorzata Kępa, Natalia Zięba, Maciej Misiołek, Rafał Wiench

**Affiliations:** 1 Department of Periodontal Diseases and Oral Mucosa Diseases, Faculty of Medical Sciences in Zabrze, Medical University of Silesia, Pl. Traugutta 2, 41-800 Zabrze, Poland; 2 Specialist Medical Practice, PolneWzgórze 11 Street, 32-300 Olkusz, Poland; 3 Municipal Hospital No. 4 in Gliwice, Zygmunta Starego 20, 44-100 Gliwice, Poland; 4 Department of Microbiology, Faculty of Pharmaceutical Sciences in Sosnowiec, Medical University of 7 Silesia in Katowice, Jagiellońska 4, 41-200 Sosnowiec, Poland; 5 Department of Otorhinolaryngology and Oncological Laryngology, Faculty of Medical Sciences in Zabrze, Medical University of Silesia in Katowice, 10 C. Skłodowskiej Street, 41-800 Zabrze, Poland

**Keywords:** antibiotics, blood platelets, growth factors, in vitro, platelet-rich fibrin

## Abstract

Platelet-rich fibrin (PRF) has emerged as a promising scaffold for drug delivery, particularly in the context of antimicrobial therapies. This systematic review evaluates the incorporation of antibiotics into PRF to determine its efficacy as a localized antimicrobial delivery system compared to plain PRF without antibiotics. A systematic review was conducted according to the Preferred Reporting Items for Systematic Reviews and Meta-Analyses (PRISMA) guidelines, including 13 in vitro studies with a moderate risk of bias. Antibiotics were incorporated into PRF using different methodologies, including systemic administration before blood collection, addition to blood before centrifugation, and injection into formed PRF matrices. Outcomes were analyzed regarding antibacterial efficacy, structural integrity of PRF, and release kinetics. Antibiotic-enhanced PRF demonstrated significant antibacterial activity against various bacterial strains. The efficacy of the enhanced PRF was dependent on the type of antibiotic, its concentration, and incorporation method. Encapsulation approaches facilitated a sustained antibiotic release, while higher antibiotic concentrations occasionally disrupted PRF integrity. Systemic administration of antibiotics before blood collection enriches PRF effectively, producing significant inhibition zones. The antibacterial effects of PRF outperformed alternative carriers, such as collagen sponges. Antibiotic-loaded PRF is a potent tool for localized antimicrobial delivery, with promising applications in clinical settings. Further research is needed to standardize preparation protocols and explore the impact of different antibiotic delivery methods on PRF’s regenerative properties.

## 1. Introduction

The utilization of platelet concentrates, including platelet-rich plasma (PRP) and platelet-rich fibrin (PRF), in regenerative medicine has been a standard practice for nearly three decades. This is due to their capacity to rapidly secrete autologous growth factors [1]. In contrast to PRP, which necessitates the incorporation of anticoagulants (e.g., bovine thrombin) during the initial phase of blood collection, PRF is obtained through centrifugation, without the use of anticoagulants, thus rendering it an exclusively autologous procedure [2]. Platelets are a vital component of the woundhealing process. Upon activation, they release a range of growth factors, including platelet-derived growth factor (PDGF), transforming growth factor (TGF-β), and insulin-like growth factor I (IGF-I) [3,4]. Among the plethora of biologically active molecules present in blood platelets, growth factors are of particular significance in the context of tissue healing and repair [5]. The evidence demonstrates that PRF exhibits a consistent and uninterrupted release of growth factors over a 10-day interval [6]. These agents exert a direct influence on the promotion of osteoblast and endothelial cell proliferation and differentiation, in addition to that of chondrocytes and various fibroblast sources [7,8]. The PRF clot forms a solid fibrin matrix, displaying a complex three-dimensional architectural structure [9]. It can thus be concluded that platelet-rich biological structures can also be employed as high-quality carriers for the targeted delivery of drugs [10]. In 2014, the development of an injectable platelet-rich fibrin (i-PRF) product was achieved through the modification of spin centrifugation forces. By centrifuging blood in non-glass tubes at reduced speeds, a flowable PRF product, designated as i-PRF, was successfully created [11,12]. The liquid PRF layer has the potential to be employed clinically for approximately 15–20 min, during which time fibrinogen and thrombin have not yet undergone conversion to a fibrin matrix [13]. Miron et al. have demonstrated that i-PRF can be employed as a drug carrier when a pharmaceutical agent is introduced into the i-PRF matrix before the onset of clot formation [14]. In a systematic review, Moraschini et al. demonstrated that all forms of PRF exhibited a notable antimicrobial activity, with a greater degree of efficacy observed against microbial pathogens compared to fungal pathogens [15]. Antimicrobial resistance poses a serious threat to global health, with significant increases in morbidity, mortality, and economic burden [16]. It is of the utmost importance that healthcare professionals, including dental surgeons, exercise judicious prescribing of antibiotics in order to stem the emergence and spread of resistance [17,18]. In 2015, it was reported that between 40% and 50% of global antibiotic prescriptions were deemed to be unwarranted [19]. As indicated in the review conducted by Teoh et al., the proportion of antibiotic prescriptions within the field of dentistry is estimated to range from 3% to 11% [20]. The direct targeting of tissues with local drug delivery strategies represents a viable approach to reducing the unnecessary use of antimicrobials [21]. The objective of this systematic review isto synthesize the existing literature on in vitro studies investigating the antimicrobial properties of PRF as a carrier for antibiotics.

## 2. Materials and Methods

This systematic review was conducted in accordance with the preferred reporting items for systematic reviews and meta-analyses (PRISMA) 2020 guidelines [22]. The protocol for this systematic umbrella review was registered in the International Prospective Register of Systematic Reviews (PROSPERO) on 29 December 2024 (PROSPERO 2024 CRD42024629278).

### 2.1. Focused Question

This systematic review was written in order to respond to the question that was posed in accordance with the PICO framework, which is the following: inin vitro studies involving platelet-rich fibrin (PRF) (population), how does the addition of antibiotics to PRF (intervention) compare to plain PRF without antibiotics (comparison) in terms of antimicrobial efficacy (outcome)?

### 2.2. Search Strategy

A comprehensive search of five electronic databases (PubMed, Scopus, Embase, Cochrane, and Google Scholar) was conducted using the appropriate syntaxes, as outlined in Table 1. The article search was conducted by two authors (W.N., R.W.) independently of one another. Furthermore, a forward/backward citation search was performed for each article that met the inclusion and exclusion criteria in order to identify additional articles related to the topic under investigation. No filters were applied in the form of a study, as these tags are added manually by the staff, and new, untagged articles may not be identified.

### 2.3. Selection of Studies

All inclusion and exclusion criteria are presented in Table 2 for reference.

**Figure 1:**
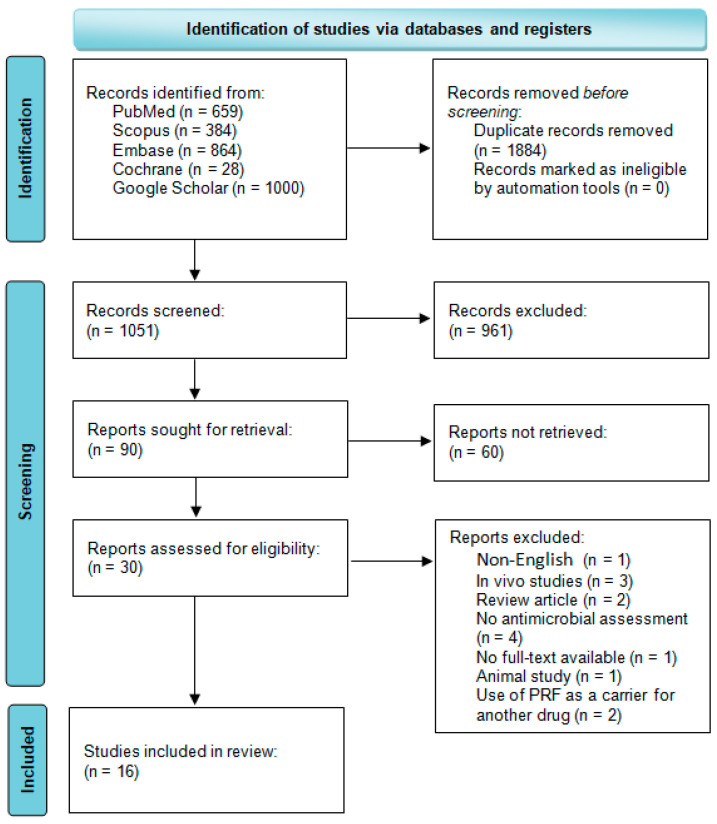
PRISMA 2020 flow diagram.

A search of the databases yielded a total of 2935 articles, of which 1051 remained after the removal of duplicates. A total of 90 reports were identified for retrieval. A total of 30 articles were subjected to an eligibility assessment. One article was excluded on the grounds of its language (other than English) [23], and four studies were identified as being in vivo. However, only three were ultimately excluded [24,25,26], as one of the studies was a two-part study, with one part conducted in vitro [27]. Four studies were excluded because they did not assess antimicrobial properties [28,29,30,31]. One article was not accessible in open access [32]. One study employed PRF derived from animal blood [33], resulting in its exclusion. Additionally, two articles were excluded due to the utilization of substances other than antibiotics in conjunction with PRF [34,35]. Two articles were not analyzed because they were review articles [10,36]. Ultimately, 16articles were subjected to analysis. All the data mentioned above are included in Figure 1.

### 2.4. Risk of Bias in Individual Studies

In the initial phase of the review selection process, each reviewer conducted an independent assessment of the titles and abstracts. This approach was adopted in order to minimize the potential influence of biases in the evaluation procedure. To quantify the level of inter-reviewer agreement, the authors employed the Cohen’s k test [37], a tool designed for this purpose. A score between 0.81 and 1.00 was deemed to indicate concordance. In instances where there was a discrepancy regarding the inclusion or exclusion of a study within the scope of the review, the respective authors engaged in deliberations until a consensus was reached.

### 2.5. Risk of Bias Across Studies Assessment

The risk-of-bias (ROB) tool for assessing in vitro studies conducted in dentistry [38] was employed to evaluate each article on an individual basis. Two authors (W.N. and R.W.) conducted the assessment of the articles independently, according to the 12 criteria described by the tool used. For each criterion, an article could receive a maximum of two points for adequately describing a parameter. One point was awarded if the issue was inadequately described. Zero points were awarded for an applicable criterion which was not addressed at all. The final score was determined by the following formula: final score = (total score × 100) = (2 × number of criteria applicable).

The scores were subsequently employed to categorize the in vitro study as exhibiting high, medium, or low ROB. The categorization was performed according to the following criteria: a score greater than 70% was indicative of a low ROB, a score of between 50% and 70% was indicative of a medium ROB, and a score of less than 50% was indicative of a high ROB.

### 2.6. Data Extraction

In addition to the data used to assess the ROB, information such as the year of publication, country in which the study was conducted, and the setting (e.g., university or other) was extracted from each article included in the review. Furthermore, data pertaining to the type of autologous platelet concentrate employed, the most crucial protocol parameters, the antibiotic utilized, the timing of its administration, the study groups, the bacterial strains involved, and the most significant outcomes were collated for articles exhibiting a low or medium ROB.

## 3. Results

### 3.1. Risk of Bias Across Studies

Of the 16 trials, three were excluded because of a high risk of bias. The remaining 13 studies had a moderate risk of bias. The criteria for which each article scored 0 includedthe provision of operator details and a detailed explanation of the sample size calculation. In addition, none of the articles used blinding at any stage. Table 3 shows the detailed risk of bias assessment for each article using the ROB tool for assessing in vitro studies conducted in dentistry [38].

### 3.2. Study Outcomes

The 13 articles included in the analysis were all conducted at universities. They were published between 2019 and 2024, with 10 of the studies published after 2022. These data are presented in Table 4.

#### 3.2.1. Types of APCs Used

The reviewed studies investigated the incorporation of various antibiotics into autologous platelet concentrates (APCs) such as leukocyte-PRF (L-PRF) [40,48], i-PRF [41,42,47], titanium-prepared PRF (T-PRF) [43], and advanced PRF+ (A-PRF+) [52].

#### 3.2.2. Antibiotics Used to Make AL-PRF

These studies explored the effects of antibiotic-enhanced PRF on bacterial inhibition and PRF’s physical and structural properties.A range of antibiotics was employed, including vancomycin [40,41], clindamycin [27,42,46], amoxicillin [48], lincomycin [39], metronidazole [46,48], amoxicillin/clavulanic acid (AMC) [27,51], gentamicin [40], linezolid [40], penicillin [45,46], doxycycline [43], ampicillin/sulbactam (SAM) [50,51,52], and a triple antibiotic paste containing ciprofloxacin, metronidazole, and minocycline [47]. The timing of antibiotic addition varied, with some studies incorporating antibiotics before centrifugation [40,45,46,48], others injecting them into the PRF matrix post-centrifugation [27,39,41,42,43,47], and some introducing antibiotics systemically before blood sampling [45,48,50,51,52]. Experimental groups generally included a control group of PRF without antibiotics, antibiotic-enhanced PRF in various forms or concentrations (e.g., solutions, powders, or encapsulated systems), and comparisons with alternative carriers such as collagen wound dressings [46] or standalone antibiotics. These setups were used to assess the effects on bacterial inhibition, PRF formation, and antibiotic release kinetics.

#### 3.2.3. Bacterial Strains Studied

The bacterial strains tested included Staphylococcus aureus [27,39,40,41,42,43,45,46,50,51,52], *Escherichia coli* [40,45,50,51,52], *Fusobacterium nucleatum* [46,48], *Enterococcus faecalis* [39,47], *Pseudomonas aeruginosa* [40,43], *Haemophilus influenzae* [40,50,51,52], *Streptococcus mitis* [40], *Sthapylococcus epidermidis* [42], and others such as *Streptococcus pneumoniae* [40,50,51,52], *Porphyromonasgingivalis* [48,50,51], *Actinomyces naeslundii* [47], and *Prevotella intermedia* [48]. 

Antibacterial activity was notably enhanced by the addition of antibiotics to PRF, with significant growth inhibition compared to control PRF across most studies. Results indicated that this activity was often dose-dependent [48] and varied among bacterial species. For instance, the highest inhibition against Actinomyces naeslundii was observed with PRF containing a triple antibiotic paste [47], while vancomycin-loaded PRF showed a complete antibacterial effect lasting for 48 h [41]. Comparisons between PRF and alternative carriers like collagen sponges demonstrated that PRF generally provides superior antibacterial effects and drug-loading capacities. For example, doxycycline-loaded T-PRF has been shown to outperform collagen in inhibiting bacterial growth [43].

#### 3.2.4. Physical Properties

The structural integrity of PRF isinfluenced by the type and concentration of the antibiotics used. While higher concentrations interfere with PRF formation, lower concentrations preserve structural properties [46]. Additionally, it has been shown that not all antibiotics can be directly used to perform AL-PRF. Bennardo et al. showed that vancomycin interferes with PRF clot formation. For this reason, it cannot be added to the blood before centrifugation [40].

Table 5 shows the data collected during the data extraction phase for each of the included articles.

## 4. Discussion

### 4.1. General Results

The collective findings of the studies indicate that the incorporation of antibiotics into APCs markedly augmenttheir antibacterial capabilities. The antibiotic-loaded PRF (AL-PRF) was observed to effectively inhibit bacterial growth across a range of bacterial strains. The antibacterial activity of AL-PRF was found to be influenced by a number of factors, including the specific antibiotic used, its concentration, and the method of incorporation. In particular, the antibacterial effect was frequently dose-dependent and exhibited variations in efficacy against different bacterial species [48]. The physical properties of the AL-PRF were generally preserved at lower antibiotic concentrations, whereas higher concentrations of certain antibiotics, such as metronidazole, resulted in the disruption of PRF formation.In the case of vancomycin, even the lowest concentration was found to impair PRF formation. It can therefore be concluded that this antibiotic is unsuitable for use in the formation of AL-PRF [40]. The utilization of encapsulated antibiotics, exemplified by vancomycin-loaded PLGA microcapsules, has illustrated the prospective for regulated and sustained drug release [41]. Nevertheless, Wang et al. conducted a study on rats in which vancomycin was incorporated into PRPwithout any microcapsules, thereby demonstrating the potential use of this additive with this APC fraction. However, the researchers demonstrated that the loaded antibiotics could reduce the concentration of growth factors released by PRP and disturb the structure of platelet–fibrin beams and the fibrin network, with a dose-dependent effect.The PRF has numerous advantages over the PRP. For instance, it does not contain anticoagulants and facilitates a more natural and efficacious woundhealing process than PRP [54]. The primary benefit of PRF is its high concentration of growth factors [55,56]. Fortunately, the lower dose of antibiotics retained its antimicrobial efficacy, while the release of growth factors fromAL-PRF, the structure of platelet–fibrin beams, and the fibrin network remained unimpaired [33]. Siawasch et al. demonstrated that, in the context of AL-PRF, the antibiotic (amoxicillin or metronidazole) does not impede the release of growth factors to any significant extent [48]. 

The release kinetics exhibited considerable variation between studies, with durations spanning from hours to several days, contingent on the antibiotic and incorporation method. The systemic administration of antibiotics prior to the collection of blood resulted in the enrichment of AL-PRF with therapeutic concentrations of antibiotics, thereby leading to a significant inhibition of bacterial growth. A comparative analysis demonstrated that PRF matrices exhibited superior antibacterial efficacy and drug-loading capacity compared to alternative carriers, such as collagen [46]. 

PRF has a variety of applications where volume preservation is required, with the optimal choice being a clot. However, in a confined space where only the treatment wound needs to be covered, PRF in the form of a membrane is the optimal solution [57,58]. Polak et al. demonstrated that there areno significant differences between the antimicrobial properties of AL-PRF in its clot form and in its membrane form [46]. The selection of an appropriate protocol for performing PRF is of significant importance with regard to future clinical outcomes [59,60]. In their study, Straub et al. compared the antimicrobial properties of different PRF centrifugation protocols after the addition of antibiotics. The highest inhibition zones were observed for the 12-min protocol, with a relative centrifugal force (RCF-max) of 652 g [50]. In a study conducted by Monika et al., the impact of incorporating metronidazole into i-PRF and C-PRF was investigated with regard to its effect on periodontal ligament fibroblast proliferation. The findings revealed that AL-PRFs containing metronidazole exhibit a higher rate of cell proliferation than i-PRF and C-PRF alone [30].

### 4.2. AL-PRF in In-Vivo Studies

At the stage of article selection, in vivo studies were excluded from the analysis as they did not conform to the PICO format. Nevertheless, the results of these studies are also relevant to the issue under discussion. As previously mentioned, the Bilginaylar et al. study was the only one that consisted of two parts: one invitro and the other in vivo. In the in vivo part, patients underwent extraction of the lower third molars. They carried out AL-PRF by injecting the antibiotic directly into the formulated PRF. The results of this study demonstrated that, despite the slightly prolonged treatment time associated with the necessity of adding an antibiotic to PRF, the VAS pain level was significantly lower, especially during the first three days after treatment, in comparison to the group that received PRF alone. For trismus, AL-PRF also exhibited significantly superior outcomes in comparison to PRF alone. The study revealed no differences in clinical parameters between the utilization of amoxicillin/clavulanic acid and clindamycin added to PRF [27].

Donmezer and Bilginaylar’s study had five groups. Patients in this study also underwent extraction of the lower third molars. The control group received only PRF. The first and third groups received PRF in the socket, with penicillin and clindamycin as oral medications. The second and fourth groups received a combination of PRF and penicillin and clindamycin in the socket. Similar to Bilginaylar et al., they carried out AL-PRF by injecting the antibiotic directly into the formulated PRF. The primary outcome variables measured were pain, swelling, analgesic intake, and trismus. There was a significant difference in the total VAS pain scores between the control and groups 3, 1, 2, and 4, in ascending order. With regard to analgesic intake, no significant difference was observed for group 1; however, statistical differences were evident between the control group and groups 2 and 3, as well as group 4. No significant differences were observed in terms of trismus and swelling among the groups.The study demonstrated that the effects of local and systemic antibiotics administered with PRF reduced postoperative outcomes. The study further demonstrated that AL-PRF enhances clinical parameters to a greater extent than PRF alone with oral antibiotic therapy [25]. 

Kadam et al. used AL-PRF in the treatment of intrabony defects. They compared PRF alone and AL-PRF with amoxicillin. The authors of this study added an amoxicillin solution to the blood before centrifugation. Clinical parameters such as probing pocket depth, relative attachment level, and relative gingival margin level showed no significant differences between groups. Only initial wound healing was better in the group with AL-PRF [24]. 

Yusri et al. compared the use of i-PRF to i-PRF with clindamycin for the surgical treatment of periodontitis using a minimally invasive surgical technique. The addition of the antibiotic to i-PRF took place after centrifugation but before clotting. Despite a significant improvement in clinical parameters, no additional benefit was demonstrated with the incorporation of clindamycin into i-PRF [26].

### 4.3. Mechanism of Antibiotic-Loaded PRF

The enhanced antibacterial efficacy of antibiotics loaded into fibrin matrices, such as PRF, can be attributed to the structural and biochemical properties of fibrin, which facilitate controlled drug release and localized antimicrobial activity. PRF provides a three-dimensional fibrin network that acts as a reservoir for antibiotic molecules, allowing for sustained drug release as the matrix gradually degrades. This controlled release mechanism prevents the rapid elimination of antibiotics, thereby maintaining therapeutic drug concentrations at the infection site for an extended period.Additionally, studies have shown that PRF can induce structural modifications in antibiotic molecules, enhancing their bioavailability and antibacterial activity. Specifically, in the case of clindamycin phosphate (CLP), Fourier-transform infrared spectroscopy analysis has indicated that PRF promotes the conversion of CLP into its more active form, clindamycin, thus increasing its efficacy against Gram-positive bacterial strains [42].

Drug release kinetics studies have demonstrated an initial burst release of approximately 80% of the encapsulated antibiotic within the first hour, followed by a gradual release over time. This immediate high-concentration exposure has been shown to effectively reduce bacterial load, while the sustained release phase ensures prolonged antimicrobial activity, thus minimizing the risk of bacterial resistance development. The findings of this study underscore the potential of PRF-based antibiotic delivery systems in enhancing localized infection control while reducing systemic side effects [42]. The incorporation of antibiotics into the fibrin structure has been demonstrated to have a significant impact on the membrane’s properties. Once the membrane is fabricated from AL-PRF, its antimicrobial properties remain evident despite the loss of exudate [46].

### 4.4. Limitations

It should be noted, however, that the included studies are subject to a number of limitations. It is notable that none of the articles included in the review were defined as having a low risk of bias. Some of the studies exhibited methodological shortcomings, which suggests that the authors may have lacked sufficient knowledge of the topic. An RCF for blood centrifugation was not provided by Egle et al. in their methodology. Furthermore, the radius of the motor in the centrifuge used was not provided. This is because the value of the centrifugal force acting on the tubes is dependent on the radius, which can be affected by the centrifuge used and the motor in it, and, therefore, RPM alone is not authoritative [61]. This constitutes not only an error in the description of the settings employed but may also have had a tangible impact on the quality of the resulting PRF utilized for the test. The observed variability between studies in the present systematic review may be attributed to the inhomogeneity of equipment used across different research centers. Recently, Takahashi et al. demonstrated that utilizing a fixed-angle centrifuge results in the accumulation of cells exclusively on the distal surface of PRF tubes, leading to the formation of membranes with an uneven distribution of cells throughout the PRF clot [62]. Furthermore, the tubes utilized for centrifugation are of significant importance [63]. Typically, glass tubes are employed for solid forms of APC, whereas plastic tubes are utilized for liquid forms. However, the majority of authors did not specify the type of tubes they used, nor whether they were coated with silica or other substances. Such data are of paramount importance with regard to the quality of the resulting APCs.

### 4.5. Clinical Implications

In light of the included articles, there are four methods for obtaining AL-PRF:

1. Blood collection in a patient who has received systemic antibiotic therapy.
2. Addition of an antibiotic to the collected blood, but before centrifugation.
3. Addition of an antibiotic to i-PRF, before clot generation.
4. Injecting the formed PRF clot with antibiotic.

Each method has its own set of advantages and disadvantages. The first method may be applicable to patients undergoing antibiotic therapy for other indications. It may also be applicable to patients with osteonecrosis of the jaws (ONJ). In these patients, systemic targeted antibiotic therapy for necrotic lesions may be less effective than for other indications. This is due to the poor blood supply to the necrotic lesions in the jaws, a phenomenon which results in a poorer distribution of the systemically administered antibiotic [64,65]. It has been revealed that bacterial colonization occurs on the exposed bone even when antibiotics are administered systemically [66]. In the context of collecting blood from patients undergoing systemic antibiotic therapy, it is essential to consider the specific antibiotic being administered. This is a crucial consideration, as different antibiotics have disparate binding pathways [67]. In their study, Straub et al. demonstrated that ampicillin/sulbactam concentrations in PRF were markedly higher than those observed with the clindamycin therapy [49]. Clindamycin is known to exhibit considerably higher plasma protein binding (approximately 60–94%) compared to ampicillin/sulbactam (approximately 20%), a fact which could account for this observation [49].

The second option for obtaining AL-PRF, involving the addition of an antibiotic to the tube prior to centrifugation, is not recommended by the authors of this review in light of current research findings. This is because it has been demonstrated that the introduction of antibiotics at the local level within the tube before centrifugation results in alterations to the physical characteristics of PRF [46]. Furthermore, Rafiee and colleagues investigated the impact of varying the timing of antibiotic addition on the release of antibiotics from AL-PRF. The researchers conducted a comparative analysis of two methods of antibiotic addition: one entailed the introduction of antibiotics to the blood sample prior to centrifugation, while the other involved the incorporation of antibiotics into the i-PRF construct following centrifugation. It was observed that the group whose blood was enriched with antibiotics prior to centrifugation was unable to sustainably release the antibiotics. In contrast, the group whose i-PRF was enriched with antibiotics after centrifugation exhibited a rapid release of all three antibiotics within the first 24 h, followed by sustained maintenance of all three antibiotics up to 14 days. Minocycline and metronidazole were still detectable in the third week [29].

The antimicrobial effects of methods 3 and 4 for obtaining AL-PRF are comparable. The incorporation of antibiotics into i-PRF may result in a more homogeneous distribution of antimicrobials upon the formation of the AL-PRF clot. Additionally, it may lead to a more homogeneous distribution of growth factors which contrasts with the significant concentration of growth factors observed at the border of the erythrocyte layer in PRF [62,68]. The indisputable benefit of AL-PRF derived from i-PRF is the capacity to obtain AL-PRF of any size [61]. Nevertheless, further research is required to provide a detailed comparison of both methods. 

Table 6 shows the advantages and disadvantages of each method for making AL-PRF.

### 4.6. Future Directions

Despite the encouraging antibacterial properties of AL-PRF, there are several challenges that require further research. A primary concern is the absence of standardized protocols for PRF preparation and antibiotic incorporation. Variations in centrifugation parameters, tube materials, and antibiotic-loading techniques can have a substantial impact on PRF’s structural integrity and drug-release kinetics. Future studies should concentrate on establishing uniform preparation guidelines to ensure reproducibility across different clinical and laboratory settings.

Another critical area for investigation is the optimization of antibiotic loading strategies, with current methods—such as systemic administration, direct incorporation into blood, or post-centrifugation injection—offering different advantages, but more research is needed to determine the most effective and biologically compatible approach. Studies should also explore the effects of antibiotic concentration on PRF’s regenerative properties to ensure that antimicrobial efficacy does not come at the expense of wound healing potential.

Furthermore, long-term in vivo studies and clinical trials are essential to validate the findings of in vitro research. While AL-PRF has demonstrated significant antibacterial activity in laboratory settings, its performance in real-world clinical applications, including periodontal regeneration and infection control, remains to be fully understood. Future research should investigate patient outcomes, healing dynamics, and potential side effects associated with AL-PRF treatment.

The integration of personalized medicine approaches holds considerable potential to enhance the efficacy of AL-PRF-based therapy. This enhancement can be realized through the meticulous tailoring of antibiotic selection and dosage, a process that is informed by patient-specific factors. These factors may include, but are not limited to, microbiome analysis, antibiotic resistance profiling, and immune response. The development of point-of-care diagnostic tools is another potential avenue for optimizing AL-PRF composition, with the ultimate objective of further improving treatment outcomes.

Finally, advancements in biomaterial engineering have the potential to further enhance PRF’s role as a drug carrier. Innovations such as nanotechnology-based drug delivery systems or bioactive modifications to the fibrin matrix could improve antibiotic retention and controlled release, rendering AL-PRF an even more effective tool for localized antimicrobial therapy. By addressing these research gaps, the clinical potential of AL-PRF can be maximized, paving the way for its widespread application in regenerative medicine and infection management.

## 5. Conclusions

The findings of this systematic review underscore the potential of antibiotic-loaded PRF as an effective localized drug delivery system with strong antibacterial properties. Antimicrobial activity depends on several factors, including the type of antibiotic, concentration, and method of incorporation into PRF. A quantity of 0.5 mL of an antibiotic, such as amoxicillin, metronidazole, or clindamycin, is sufficient to achieve antimicrobial properties without affecting the physical properties of PRF. In patients who are not receiving systemic antibiotic therapy, the optimal approach is to incorporate the antibiotic into the PRF/i-PRF only after centrifugation. Compared to other carriers such as collagen, PRF consistently showed superior antibacterial effects and drug-loading capacity. These properties make PRF an innovative and promising scaffold for targeted antimicrobial therapies in various clinical contexts. However, careful consideration must be given to the preparation protocols to optimize both antimicrobial efficacy and structural integrity. Further studies are required to accurately assess the relative merits of different protocols, in order to determine which, if any, is the most efficacious.

## Figures and Tables

**Table 1:** Search terms given for individual databases with limitations and number of results.

Database	Search Terms	Limitations	Results
PubMed	(“Blood Platelets” [Mesh] OR “Platelet-Rich Plasma” [Mesh] OR “Platelet-Rich Fibrin” [Mesh]) AND (“Antibiotic Prophylaxis” [Mesh] OR “Anti-Bacterial Agents” [Pharmacological Action] OR “Anti-Bacterial Agents” [Mesh])	Years 2000–2024.	659
Scopus	(“Blood Platelets” OR “Platelet-Rich Plasma” OR “Platelet-Rich Fibrin”) AND (“Antibiotic Prophylaxis” OR “Anti-Bacterial Agents” OR “Antibacterial Agents”)	Years 2000–2024.	384
Embase	(‘blood platelet’/exp OR ‘platelet rich plasma’/exp OR ‘platelet rich fibrin’/exp OR ‘blood platelets’ OR ‘prp’ OR ‘platelet concentrate’) AND (‘antibiotic prophylaxis’/exp OR ‘antibacterial agents’ OR ‘antibiotic prophylaxis’ OR ‘antibacterials’ OR ‘antibiotic therapy’) AND [2000–2024]/py	Years 2000–2024.	864
Cochrane	(“Blood Platelets” OR “Platelet-Rich Plasma” OR “Platelet-Rich Fibrin”) AND (“Antibiotic Prophylaxis” OR “Anti-Bacterial Agents” OR “Antibacterial Agents”)	No limitations.	28
Google Scholar	(“Blood Platelets” OR “Platelet-Rich Plasma” OR “PRP” OR “Platelet-Rich Fibrin” OR “PRF”) AND (“Antibiotic Prophylaxis” OR “Anti-Bacterial Agents” OR “Antibacterial Agents”)	Search limited to the 1000 most accurate results between 2000 and 2024.	5850

**Table 2:** Selection criteria for papers included in the systematic review.

Inclusion Criteria	Exclusion Criteria
In vitro studies Low or moderate risk of bias Antimicrobial assessment Human studies Full-text available English language	Narrative reviews Systematic reviews Meta-analysis Non-English language publications High risk of bias of the study Letters to the Editor Grey literature Conference papers Animal studies In vivo studies Use of PRF as a carrier for another drug

**Table 3:** The results of the quality assessment and risk of bias across the studies.

Criterion Number	Study															
	Alhaffar et al. (2022) [39]	Bennardo et al. (2023) [40]	Bilginaylar et al. (2024) [27]	Dubnika et al. (2021) [41]	Egle et al. (2022) [42]	Ercan et al. (2022) [43]	Knafl et al. (2017) [44]	Ozcan et al. (2024) [45]	Polak et al. (2019) [46]	Rafiee et al. (2020) [47]	Siawasch et al. (2022) [48]	Straub et al. (2024) [49]	Straub et al. (2023) [50]	Straub et al. (2024) [51]	Straub et al. (2022) [52]	Walianto et al. (2022) [53]
I	2	2	1	2	2	2	2	2	2	2	2	1	2	2	1	0
II	0	0	0	0	0	0	0	0	0	0	0	0	0	0	0	0
III	2	2	2	2	1	1	1	2	1	0	2	0	2	2	2	0
IV	2	2	2	2	2	2	2	2	2	2	2	1	2	2	N/A	2
V	1	2	2	2	2	2	1	2	2	1	2	2	2	2	2	2
VI	0	0	0	0	0	0	0	0	0	0	0	0	0	0	0	0
VII	0	0	0	0	0	0	0	0	0	2	0	0	0	0	N/A	0
VIII	2	2	1	2	2	2	1	2	2	2	2	2	2	2	2	1
IX	1	0	0	0	0	0	0	0	0	0	0	0	0	0	0	0
X	0	0	0	0	0	0	0	0	0	0	0	0	0	0	0	0
XI	2	2	2	2	2	2	2	2	2	2	2	2	2	2	2	1
XII	2	2	2	2	2	2	1	2	2	2	2	2	2	2	2	1
Score	58.3%	58.3%	50%	58.3%	54.2%	54.2%	41.7%	58.3%	54.2%	54.2%	58.3%	41.7%	58.3%	58.3%	55%	29.2%
Risk of bias	Medium	Medium	Medium	Medium	Medium	Medium	High	Medium	Medium	Medium	Medium	High	Medium	Medium	Medium	High

I—clearly stated aims/objectives, II—detailed explanation of sample size calculation, III—detailed explanation of sampling technique, IV—details of comparison group, V—detailed explanation of methodology, VI—operator details, VII—randomization, VIII—method of measurement of outcome, IX—outcome assessor details, X—blinding, XI—statistical analysis, XII—presentation of results, 2—adequately specified, 1—inadequately specified, 0—not specified, N/A—not applicable.

**Table 4:** A general overview of the studies.

Author	Year	Country	Setting
Alhaffar et al. [39]	2022	Syria	University
Bennardo et al. [40]	2023	Italy	University
Bilginaylar et al. [27]	2024	Turkey	University
Dubnika et al. [41]	2021	Latvia	University
Egle et al. [42]	2022	Latvia	University
Ercan et al. [43]	2022	Turkey	University
Ozcan et al. [45]	2024	Turkey	University
Polak et al. [46]	2019	Israel	University
Rafiee et al. [47]	2020	Iran	University
Siawasch et al. [48]	2022	Belgium	University
Straub et al. [50]	2023	Germany	University
Straub et al. [51]	2024	Germany	University
Straub et al. [52]	2022	Germany	University

**Table 5:** Detailed characteristics of the in vitro studies included in this review.

Author/Year	APC Used	Antibiotics Used	When and How Antibiotic Was Added	Study Groups	Bacterial Strains Used/Culture Media Used	Results
Alhaffar et al. (2022) [39]	PRF (3000 rpm, 10 min, 400 g) Plastic tubes were used to delay PRF formation	Lincomycin 600 mg	Locally injected/mixed into PRF After centrifugation but before clot formation	1. Control group—PRF without antibiotics PRF with Lincomycin in the form of: 2. Ampoule 0.5 mL 3. Ampoule 1 ml 4. Solution 0.5 mL 5. Solution 1 mL 6. Powder 50 mg 7.Powder 100 mg	*S. aureus* *E. faecalis* Culture medium: Mueller–Hinton agar	Only the powder disrupted clot formation. The best results for bacterial growth inhibition and physical properties were in group 2. Bacterial inhibition lasted for 10 days in culture.
Bennardo et al. (2023) [40]	L-PRF (2700 rpm, 12 min, 710 g)	Gentamicin 1 mg/mL Linezolid 2 mg/mL Vancomycin 5 mg/mL	Locally injected/mixed into PRF Before centrifugation	1. Control group—PRF without antibiotics PRF with: 2–5. Gentamicin 0.25, 0.5, 0.75, 1 mL 6–9. Linezolid 0.25, 0.5, 0.75, 1 mL 10–13. Vancomycin 0.25, 0.5, 0.75, 1 mL	*P. aeruginosa* *S. pneumoniae* *S. mitis* *H. influenzae* *E. coli* *S. aureus* Culture media: chocolate agar for *H. Influenzae*, Columbia agar for the rest	Vancomycin interfered with PRF formation. Gentamicin and linezolid did not affect PRF and were released from membranes within the time examined (4 days). The control PRF had a slight antibacterial activity against all tested microorganisms. Gentamicin-PRF was very effective against all tested microorganisms. The results for linezolid-PRF were similar, except that it was as effective as the control PRF against *E. coli* and *P. aeruginosa*.
Bilginaylar et al. (2024) [27]	PRF (3000 rpm, 10 min, 700 g)	Amoxicillin with clavulanic acid (1000/200 mg) Clindamycin (600 mg/4 mL)	After centrifugation Injection into the centre of the PRF matrix	1. Control group—PRF without antibiotics PRF with injected: 2 and 3. 0.5 mL and 1 mL AMC (1000/200 mg) 4 and 5. 0.5 mL and 1 mL clindamycin (600 mg/4 mL)	*S. aureus* Culture medium: blood agar	PRF preparations containing AMC or clindamycin significantly inhibited the growth of s. aureus bacteria. No significant differences were identified between groups 2 and 3, or between groups 4 and 5. However, it was demonstrated that both groups 4 and 5 exhibited a significantly greater inhibition of bacterial growth in comparison to groups 2 and 3.
Dubnika et al. (2021) [41]	i-PRF (700 rpm, 3 min, motor radius 100 mm)	Vancomycin	Locally injected/mixed into PRF After centrifugation but before clot formation	1. Control group—PRF without antibiotics 2. PRF with VANKA-loaded liposomes 3. PRF with VANKA- added as free drug powder, non-encapsulated 4. PRF with VANKA loaded PLGA microcapsules	*S. aureus* Culture medium: Mueller–Hinton agar	It is not possible to homogenously mix liposomes into the PRF matrix. The utilization of a carrier system can facilitate the regulated release of VANKA for a period of six to ten days. The complete antibacterial effect was observed to last for 48 h. No antibacterial properties were observed in samples lacking VANKA. The utilization of VANKA in PRF scaffolds devoid of a carrier system does not guarantee the regulated delivery of the active VANKA form at the therapeutic effect level.
Egle et al. (2022) [42]	i-PRF Men: 700 rpm 6 min Women: 700 rpm 5 min	Clindamycin	Locally injected/mixed into PRF After the centrifugation	1. Control group—PRF without antibiotics 2. PRF with clindamycin 3. Clindamycin	*S. aureus* *S. epidermidis* Culture medium: Mueller–Hinton broth	CLP, in combination with PRF, showed a stronger antibacterial activity against *S. aureus* and *S. epidermidis* compared to pure CLP solution.
Ercan et al. (2022) [43]	T-PRF (2700 rpm, 12 min, 635 g) using T-PRF tubes	Doxycycline 2 mL of doxycycline solution at 3 mg/mL	Locally injected/mixed into PRF After the centrifugation	1. Control group—T-PRF without antibiotics 2. T-PRF with doxycycline 3. Collagen with doxycycline	*S. aureus* *P. aeruginosa* Culture media: nutrient broth and nutrient agar	In comparison tocollagen, approximately sevenfold more doxycycline was loaded into T-PRF. It was observed that 25% of the loaded doxycycline was released from T-PRF in comparison to only 12% from collagen within 72 h. The largest inhibition zone diameter was observed for T-PRF/doxycycline.The collagen/doxycycline group demonstrated no inhibition zone. The T-PRF/doxycycline group displayed a dense fibrin structure on SEM images, in contrast to the T-PRF group.
Ozcan et al. (2024) [45]	PRF (2700 rpm, 12 min, 708 g)	Penicillin	Before the centrifugation 1. Local administration to the blood 2. Systemic administration	1. Control group—PRF without antibiotics 2. PRF with the local addition of 0.2 mL antibiotic 3. PRF from the blood of patients given 2 g of penicillin one hour earlier	*S. aureus* *E. coli* Culture medium: Mueller–Hinton agar	No *S. aureus* growth was observed at any time point (24, 48, 72 h) in the twogroups, resulting in a bacterial count of zero. In contrast, *S. aureus* growth was observed in groups 1 and 2 throughout all 3 days. Although group 3 exhibited less bacterial growth compared to group 1, no statistical significance was observed between these two groups. The same results were obtained for *E. coli*
Polak et al. (2019) [46]	PRF (2700 rpm, 12 min, 735 g)	Metronidazole 5 mg/mL Clindamycin 150 mg/mL Penicillin 1 mU/mL	Locally injected/mixed into PRF Before the centrifugation	Collagen wound dressing with: 1. 0.5 mL MET 2. 0.5 mL Clindamycin 3. 0.5 mL Penicillin 4. 0.5 mL saline PRF with: 5. 0.5 mL MET 6. 0.5 mL Clindamycin 7. 0.5 mL Penicillin 8. 0.5 mL saline	*S. aureus* *F. nucleatum* Culture medium: blood agar	The incorporation of antibiotic solutions at concentrations of 2 or 1 mL resulted in notable alterations to the physical characteristics of PRF, whereas the introduction of a 0.5 mL solution did not elicit comparable effects. The addition of saline to PRF resulted in only minimal antibacterial activity, whereas all PRFs containing antibiotics demonstrated a significant antibacterial effect. No notable differences were observed between the raw (clot) and the pressed (membrane) forms of PRF. The anti-bacterial properties of collagen sponges with and without antibiotics were comparable to those of PRF. These properties were maintained for up to four days after preparation.
Rafiee et al. (2020) [47]	i-PRF (no data about rpm, 3 min, 60 g)	A triple antibiotic paste containing ciprofloxacin, metronidazole and minocycline with the concentration of 1 mg/mL	Locally injected/mixed into PRF After the centrifugation 2 mL of i-PRF with 2 mL of stock solution of triple antibiotic mixture	Group 1: triple antibiotic mixture Group 2: I-PRF containing triple antibiotic mixture, and Group 3: antibiotic-free I-PRF scaffold	*A. naeslundii* *E. faecalis* Culture media: no data	The highest antibacterial activity against *A. naeslundii* was observed in group 2. However, groups 1 and 2 demonstrated comparable antibacterial properties against *E. faecalis.* Overall, the groups exhibited higher levels of antibacterial activity against *E. faecalis* than against *A. naeslundii.* Notably, group 2 was capable of significantly reducing the number of live bacteria to nearly 92%.
Siawasch et al. (2022) [48]	L-PRF (2700 rpm, 12 min, 408 g)	Metronidazole 5 mg/mL Amoxicillin	Before the centrifugation 1. local administration to the blood 2. systemic administration	1. Control group—L-PRF without antibiotics 2. L-PRF with 0.125 mL MET 3. L-PRF with 0.25 mL MET 4. L-PRF with 0.5 mL MET 5. L-PRF after systemic MET 500 mg 6. L-PRF after systemic amoxicillin 2 g	*P. gingivalis* *P. intermedia* *F. nucleatum* Culture medium: blood agar	The release of metronidazole was detectable up to day 3, with the highest concentration occurring during the first 4 h. The antibacterial effect was most pronounced against *P. gingivalis* and least pronounced against *P. intermedia.* Groups 2, 3, 4 resulted in a significant increase in the inhibition distance when compared to group 1. The inhibitory effect of L-PRF membranes modified with a MET solution was found to be dose-dependent. The consumption of amoxicillin resulted in a significantly higher inhibition distance against all three pathogens. Following the administration of MET, a notable increase in the inhibition distance was observed against *P. gingivalis* and *F. nucleatum*, but not against *P. intermedia.*
Straub et al. (2023) [50]	PRF A. 1300 rpm, 8 min, 208 g B. 2300 rpm, 12 min, 652 g C. 1500 rpm, 14 min, 276 g	Ampicillin/sulbactam	Systemic administration intravenously (2 g ampicilin 1 g sulbactam)	1. PRF formed under protocol A without antibiotics 2. PRF formed under protocol A 3. PRF formed under protocol B 4. PRF formed under protocol C	*E. coli* *H. influenzae* *S. pneumoniae* *S. aureus* *P. gingivalis* Culture media: Brucella blood agar for *P. gingivalis*, Mueller–Hinton agar for the rest	A single dose of SAM is sufficient to reach high concentrations ofPRF in all protocols (150 μg/mL), comparable to the plasma SAM concentration. The inhibition zones observed in the *E. coli* cultures were found to be statistically significant, with protocol B resulting in the largest zones. The same results were observed when the PRF discs were stored for 24 h at 36 °C prior to testing. The PRF discs obtained from protocol B also caused the largest inhibition zones. The results were found to be statistically significant for *E. coli*, *H. influenzae*, and *P. gingivalis*.
Straub et al. (2024) [51]	PRF (2300 rpm, 12 min, 652 g)	Ampicillin/sulbactam Amoxicillin/clavulanic acid	Systemic administration intravenously (2 g ampicilin, 1 g sulbactam) 15–25 min before blood sampling Oral therapy of AMC (875/125 mg or 1750/250 mg) One hour before blood sampling	1. Control group—PRF without antibiotics 2. PRF after oral therapy with AMC, 1 g 3. PRF after oral therapy with AMC, 2 g 4. PRF after intravenous therapy with SAM, 3 g	*E. coli* *H. influenzae* *S. pneumoniae* *S. aureus* *P. gingivalis* Culture media: Brucella blood agar for *P. gingivalis* Mueller–Hinton agar for the rest	The inhibition zones for fresh PRF and the single oral dose of AMC were 0.0, 4.7, 15.2, 2.3, and 0.9 mm (for *E. coli*, *S. aureus*, *S. pneumoniae*, *H. influenzae*, and *P. gingivalis*, respectively). For the double oral dose, the values were 0.0, 11.4, 20.0, 8.1, and 7.4 mm. The IZs for SAM were 11.9, 18.2, 24.7, 20.3, and 22.1 mm. There were significant differences between the parenteral and oral applications, as well as between the different oral doses.
Straub et al. (2022) [52]	A-PRF+ (1300 rpm, 8 min, 208 g)	Ampicillin/sulbactam	Systemic administration intravenously (2 g ampicillin, 1 g sulbactam) At least threedoses and an additional dose 10 min before blood sampling	1. Control group—PRF without antibiotics 2. AMC 3. PRF after systemic therapy with AMC	*E. coli* *H. influenzae* *S. pneumoniae* *S. aureus* Culture media: Mueller–Hinton agar	The PRF is characterized by a high level of enrichment with SAM, which is subsequently released into the surrounding environment. The concentration of the antibiotic in PRF was found to be comparable to that of ampicillin/sulbactam in plasma. The IZ of PRF was found to be comparable to that of the standard SAM discs employed in sensitivity testing.

APC—autologous platelet concentrates, CLP—clindamycin phosphate, PRF—platelet-rich fibrin, rpm—revolutions per minute, VANKA—vancomycin hydrochloride, PLGA—poly lactic-co-glycolic acid, i-PRF—injectable platelet-rich fibrin, L-PRF—leukocyte-platelet-rich fibrin, T-PRF—titanium-prepared platelet-rich fibrin, SEM—scanning electron microscope, MET—metronidazole, SAM—ampicillin/sulbactam, AMC—amoxicillin/clavulanic acid, IZ—inhibition zone.

**Table 6:** Advantages and disadvantages of each method of making AL-PRF.

Method of Obtaining AL-PRF	Advantages	Disadvantages
Blood collection in a patient who has received systemic antibiotic therapy	- Ensures antibiotic incorporation into PRF without altering its physical properties. - Particularly useful for patients already undergoing antibiotic therapy, especially those with ONJ. - Systemic antibiotics may reach necrotic lesions with limited vascularization.	- Limited to patients already receiving systemic antibiotics. - Variability in antibiotic binding to plasma proteins affects antibiotic concentration in PRF. - Effectiveness depends on the specific antibiotic used.
Adding an antibiotic to the collected blood, but before centrifugation	- Theoretically allows for uniform distribution of antibiotics within AL-PRF.	- Alterations in PRF’s physical properties due to antibiotic interaction. - Reduced ability to sustain antibiotic release over time. - Not recommended based on current research findings.
Addition of antibiotic to i-PRF, before clot generation	- Enables homogeneous distribution of antibiotics and growth factors. - Ensures sustained antibiotic release. - Allows for obtaining AL-PRF of any desired size.	- Necessity to wait for a clot to form from i-PRF.
Injecting the formed PRF clot with antibiotic	- Simple and effective method. - Ensures localized delivery without affecting PRF formation.	- Potentially less homogeneous distribution of the antibiotic within the clot. - Risk of non-uniform antibiotic release kinetics.

PRF—platelet-rich fibrin, i-PRF—injectable platelet-rich fibrin, AL-PRF—antibiotic-loaded platelet-rich fibrin, ONJ—osteonecrosis of the jaw.
